# Prediction of lymph node metastasis by tumor-infiltrating lymphocytes in T1 breast cancer

**DOI:** 10.1186/s12885-020-07101-y

**Published:** 2020-06-26

**Authors:** Koji Takada, Shinichiro Kashiwagi, Yuka Asano, Wataru Goto, Rika Kouhashi, Akimichi Yabumoto, Tamami Morisaki, Masatsune Shibutani, Tsutomu Takashima, Hisakazu Fujita, Kosei Hirakawa, Masaichi Ohira

**Affiliations:** 1 Department of Breast and Endocrine Surgery, Osaka City University Graduate School of Medicine, 1-4-3 Asahi-machi, Abeno-ku, Osaka, 545-8585 Japan; 2 Department of Gastrointestinal Surgery, Osaka City University Graduate School of Medicine, 1-4-3 Asahi-machi, Abeno-ku, Osaka, 545-8585 Japan; 3 Department of Scientific and Linguistic Fundamentals of Nursing, Osaka City University Graduate School of Nursing, 1-5-17 Asahi-machi, Abeno-ku, Osaka, 545-0051 Japan

**Keywords:** Breast cancer, Tumor-infiltrating lymphocytes, Tumor immune-microenvironment, Lymph node metastasis, Sentinel lymph node

## Abstract

**Background:**

Lymph node metastasis is more likely in early-stage breast cancer with lower tumor-infiltrating lymphocyte (TIL) density. Therefore, we investigated the correlation between TILs and lymph node metastasis in cT1 breast cancer patients undergoing surgery and the usefulness of TILs in predicting sentinel lymph node metastasis (SLNM) in cT1N0M0 breast cancer.

**Methods:**

We investigated 332 breast cancer patients who underwent surgery as the first-line treatment after preoperative diagnosis of cT1. A positive diagnosis of SLNM as an indication for axillary clearance was defined as macrometastasis in the sentinel lymph node (SLN) (macrometastasis: tumor diameter > 2 mm). Semi-quantitative evaluation of lymphocytes infiltrating the peritumoral stroma as TILs in primary tumor biopsy specimens prior to treatment was conducted.

**Results:**

For SLN biopsy (SLNB), a median of 2 (range, 1–8) SLNs were pathologically evaluated. Sixty cases (19.4%) of SLNM (macrometastasis: 46, micrometastasis: 16) were observed. Metastasis was significantly greater in breast cancers with tumor diameter > 10 mm than in those with diameter ≤ 10 mm (p = 0.016). Metastasis was significantly associated with lymphatic invasion (p < 0.001). These two clinicopathological factors correlated with SLNM even in patients diagnosed with cN0 (tumor size; p = 0.017, lymphatic invasion; p = 0.002). Multivariate analysis for SLNM predictors revealed lymphatic invasion (p = 0.008, odds ratio [OR] = 2.522) and TILs (p < 0.001, OR = 0.137) as independent factors.

**Conclusions:**

Our results suggest a correlation between lymph node metastasis and tumor immune-microenvironment in cT1 breast cancer. TIL density may be a predictor of SLNM in breast cancer without lymph node metastasis on preoperative imaging.

## Background

Breast cancer frequently metastasizes to the axillary lymph nodes, and the status of axillary lymph nodes metastasis is a prognostic factor in early breast cancer. Sentinel lymph node (SLN) biopsy (SLNB) is commonly used for pathological evaluation even if axillary lymph node metastasis is not detected on imaging. SLNB is considered a minimally invasive method based on the results of previously reported randomized controlled trials [1, 2]. However, in recent years, SLNB is being considered excessively invasive for breast cancer patients with a small primary tumor because it is unlikely to have metastasized [3]. Therefore, clinical trials that omit SLNB for cN0 breast cancer patients diagnosed by ultrasonography (US) are underway [4, 5]. One of the prospective randomized trials targeted cT1 breast cancer patients and the other trial targeted small primary tumor that could be resected with breast-conserving surgery. However, to summarize the previous reports, the SLN metastasis (SLNM) rate in T1 breast cancer was 18.8–29.6%, which is substantial [6–10]. These studies have additionally reported various predictors of SLNM.

The tumor microenvironment, comprising cancer-associated fibroblastic cells, angiogenic vascular cells, and infiltrating immune cells, is strongly involved in cancer invasion and metastasis [11, 12]. Among these cells, lymphocytes around tumors, the so-called “tumor-infiltrating lymphocytes (TILs)”, are used as a simple indicator of tumor-related immune response. It has been suggested that TILs may also affect cancer invasion and metastasis [11]. However, in breast cancer, TILs are strongly affected by the subtype of breast cancer. Hormone receptor-negative breast cancers such as human epidermal growth factor receptor 2 (HER2)-enriched breast cancer (HER2-enriched BC) and triple-negative breast cancer (TNBC) are known to have higher TIL density than hormone receptor-positive breast cancers [13, 14].

Therefore, we hypothesized that lymph node metastasis is likely to occur in breast cancer with lower TIL density. If this hypothesis is correct, we can also hypothesize that TILs could be a predictor of SLNM. Since the tumor size is a strong predictor of SLNM, and a prospective randomized trial that omit SLNB for cT1N0 breast cancer patients is in progress, we investigated the correlation between TILs and lymph node metastasis in cT1 breast cancer patients undergoing surgery along with the usefulness of TILs in predicting SLNM for cT1N0M0 breast cancer in this study.

## Methods

### Patients

In this study, we included 332 breast cancer patients who had undergone surgery as the first-line treatment after preoperative diagnosis of cT1 from April 2007 to October 2015 at Osaka City University Hospital. In all patients, breast cancer was diagnosed pathologically by core-needle biopsy (CNB) or vacuum-assisted biopsy (VAB). The expressions of estrogen receptor (ER), progesterone receptor (PgR), HER2, and Ki67 in the biopsy tissue was determined immunohistologically. Subsequently, we classified breast cancer based on the results of immunohistological staining as follows: HER2-enriched BC (ER-, PgR-, and HER2+); TNBC (negative for ER, PgR, and HER2); hormone receptor (HR) + HER2 + BC (hormone receptor and HER2-positive breast cancer; ER+ and/or PgR+, and HER2+); and HR + HER2-BC (hormone receptor-positive and HER2-negative breast cancer; ER+ and/or PgR+, and HER2-). Based on previous reports, the cutoff value for Ki67 was considered to be 14% [15]. US, computed tomography (CT), and bone scintigraphy were performed to rule out distant metastasis. All patients underwent mastectomy or breast-conserving surgery. In patients in whom axillary lymph node metastasis was suspected on imaging, axillary lymph node dissection was performed. In contrast, in patients in whom metastasis to the lymph nodes was not suspected, SLNB was performed. The SLN was identified using a combination of radioisotope and dye methods, as per previous reports [16, 17]. SLNs were sliced into 2-mm-thick slices and pathologically examined for metastases [18, 19]. SLNM was classified according to previous reports; (Macrometastasis: tumor diameter > 2 mm. Micrometastasis: tumor diameter > 0.2 mm, ≤2 mm, or < 200 tumor cells. Isolated tumor cells: tumor diameter < 0.2 mm or < 200 tumor cells) [20].

### Histopathological evaluation of TIL density

**Fig. 1:**
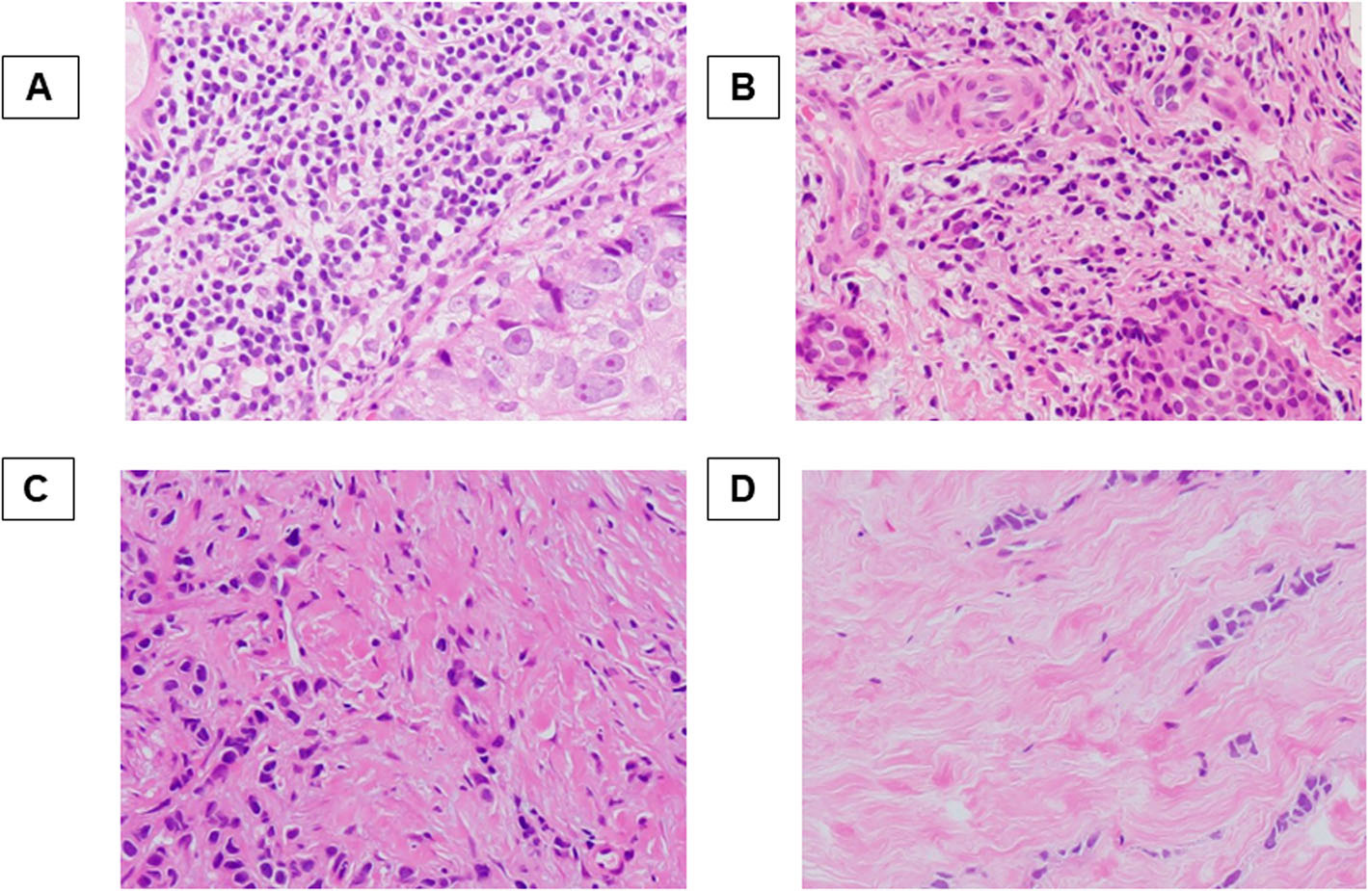
Histopathologic analysis for tumor-infiltrating lymphocyte (TIL) density was performed on a single full-face hematoxylin and eosin-stained tumor section. TIL density scores were defined as 3, 2, 1, and 0 if the area of stroma with lymphoplasmacytic infiltration around the invasive tumor cell nests was > 50% (**a**); > 10–50% (**b**); ≤10% (**c**); and absent (**d**), respectively

Histopathological evaluation of TIL density was performed in the biopsy specimens. The definition and evaluation of TIL were based on the International TILs working group 2014 guideline, which calculates the average density of the infiltrating lymphocytes within the tumor stroma in five randomly selected fields [21]. We defined 4 classes or scores according to TIL density according to previous reports; (score 3; > 50%, score 2; > 10–50%, score 1; ≤10%, or score 0; absent) (Fig. 1) [22, 23].

### Statistical analysis

Statistical analyses were performed using JMP software package (SAS, Tokyo, Japan). To compare the distribution of TIL density according to the state of lymph node metastasis, we performed Student’s t test. Pearson’s chi-square test was used to evaluate the correlation between two groups based on clinicopathological features. Odds ratios (ORs) and 95% confidence intervals (CIs) were calculated using logistic regression analysis. Multivariable analysis was performed using the multivariable logistic regression model. P-values less than 0.05 were considered significant.

### Ethics statement

This study was conducted at Osaka City University, Osaka, Japan, and conducted in accordance with the Declaration of Helsinki. The study protocol was approved by the Ethics Committee of Osaka City University (approve number: #926). All patients were informed of the investigational nature of this study and provided their written, informed consent.

## Results

### Clinicopathological features

Table 1 shows the clinicopathological features of 332 patients with cT1N0-2 M0 breast cancer who underwent surgery and 319 patients with cT1N0M0 breast cancer who underwent SLNB. Therefore, 13 patients (3.9%) were diagnosed with axillary lymph node metastases on imaging investigation (cN1: 11 patients (3.3%), cN2: 2 patients(0.6%)).In both groups, the median age was 59 (range, 29–79) years, and the median tumor diameter was 13 mm (range, 4.0–20.0 mm). In patients with cT1N0M0 breast cancer, 262 patients (82.1%) were positive for ER, 194 (60.8%) were positive for PgR, and 24 (7.5%) were positive for HER2. High Ki67 expression was observed in 123 patients (38.8%). The following results were demonstrated by the intrinsic subtypes: HR + HER2-BC: 255 patients (79.9%), HR + HER2 + BC: 10 patients (3.1%), HER2-enriched BC 14 patients (4.4%), TNBC: 40 patients (12.5%). Pathologically, lymphatic invasion was observed in 95 patients (29.8%), and venous invasion in 13 patients (4.1%). Regarding the nuclear grade, only 36 patients (11.3%) were diagnosed with grade 3. These results did not differ significantly when compared with the entire group of cT1 patients undergoing surgery.

**Table 1:** Clinicopathological features of 332 patients who had surgery after being diagnosed with cT1N0-2 M0 breast cancer, including 319 cT1N0M0 breast cancer

Parameters	Number of all patients (*n* = 332) (%)	Number of cN0 patients (*n* = 319) (%)
Age at operation (years old)	median 59 (range, 29–79)	median 59 (range, 29–79)
Tumor size (mm)	median 13 (range, 4–20)	median 13 (range, 4–20)
Clinical lymph node metastasis cN0 / cN1 / cN2	319 (96.1%) / 11 (3.3%) / 2 (0.6%)	–
Estrogen receptor Negative / Positive	59 (17.8%) / 273 (82.2%)	57 (17.9%) / 262 (82.1%)
Progesterone receptor Negative / Positive	130 (39.2%) / 202 (60.8%)	125 (39.2%) / 194 (60.8%)
HER2 Negative / Positive	306 (92.2%) / 26 (7.8%)	295 (92.5%) / 24 (7.5%)
Ki67 ≤ 14% / > 14%	206 (62.0%) / 126 (38.0%)	196 (61.4%) / 123 (38.6%)
Intrinsic subtype HR + HER2-BC / HR + HER2 + BC / HER2enriched BC / TNBC	265 (79.8%) / 11 (3.3%) / 15	255 (79.9%) / 10 (3.1%) / 14
	(4.5%) / 41 (12.4%)	(4.4%) / 40 (12.6%)
Lymphatic invasion ly0 / ly1	229 (69.0%) / 103 (31.0%)	224 (70.2%) / 95 (29.8%)
Venous invasion v0 / v1	318 (95.8%) / 14 (4.2%)	306 (95.9%) / 13 (4.1%)
Nuclear grade 1 / 2 / 3	164 (49.4%) / 129 (38.9%) / 39	158 (49.5%) / 125 (39.2%) / 36
	(11.7%)	(11.3%)
Pathological lymph node metastasis pN0 / pN1mic / pN1a / pN2	257 (77.4%) / 16 (4.8%) / 54	257 (80.6%) / 16 (5.0%) / 46
	(16.3%) / 5 (1.5%)	(14.4%) / 0 (0.0%)
TILs (score) 0 / 1 / 2 / 3	29 (8.7%) / 243 (73.2%) / 57	25 (7.8%) / 235 (73.7%) / 56
	(17.2%) / 3 (0.9%)	(17.6%) / 3 (0.9%)

HER2: human epidermal growth factor receptor 2. HR + HER2-BC: hormone receptor-positive and HER2 negative breast cancer (ER+ and/or PgR+, and HER2-). HR + HER2 + BC: hormone receptor-positive and HER2 positive breast cancer (ER+ and/or PgR+, and HER2+). HER2 enriched BC: human epidermal growth factor receptor 2-enriched breast cancer (ER-, PgR-, and HER2+). TNBC: triple negative breast cancer (ER-, PgR-, and HER2-). TILs: tumor- infiltrating lymphocytes

For SLNB, a median of 2 (range, 1–8) SLNs were identified and evaluated pathologically. There were 60 cases (19.4%) of SLNM (macrometastasis: 46 cases, micrometastasis: 16 cases). The intrinsic subtype of all breast cancers with micrometastasis was HR + HER2-BC. All patients who underwent axillary dissection due to lymph node metastasis on radiological examination had pathological metastasis to the lymph nodes.

When TIL densities were examined in the biopsied tissues, in cN0 cases, 25 patients (7.8%) had score 0, 235 (73.7%) had score 1, 56 (17.6%) had score 2, and three (0.9%) had score 3. In the 13 cases in which lymph node metastasis was detected by imaging, four patients had score 0, eight had score 1, and one had score 2.

### Correlation between clinicopathological features and lymph node metastasis

The correlations between clinicopathological features and lymph node metastasis are listed in Table 2. Metastasis was significantly higher in breast cancers with tumor diameter > 10 mm than in those with diameter ≤ 10 mm (p = 0.016). Additionally, metastasis was significantly associated with lymphatic invasion (p < 0.001). These two clinicopathological factors correlated with SLNM even in patients diagnosed with cN0 (tumor size; p = 0.017, lymphatic invasion; p = 0.002) (Table 3).

**Table 2:** Correlation between lymph node metastasis and clinicopathological features in cT1 breast cancer patients undergoing surgery

Parameters	All intrinsic subtype (*n* = 332)			HR + HER2-BC (*n* = 265)			HR + HER2 + BC (*n* = 11)			HER2enriched BC (*n* = 15)			TNBC (*n* = 41)		
	pN0 or 1mic (*n* = 273)	pN1a or 2 (*n* = 59)	*p* value	pN0 or 1mic (*n* = 217)	pN1a or 2 (*n* = 48)	*p* value	pN0 (*n* = 9)	pN1a or 2 (*n* = 2)	*p* value	pN0 (*n* = 11)	pN1a or 2 (*n* = 4)	*p* value	pN0 (*n* = 36)	pN1a or 2 (*n* = 5)	*p* value
Age (years old)															
≤ 60	158 (57.9%)	28 (47.5%)	0.144	130.(59.9%)	23.(47.9%)	0.128	5 (55.6%)	1 (50.0%)	0.887	7(63.6%)	3 (75.0%)	0.680	16 (44.4)	1 (20.0%)	0.299
> 60	115 (42.1%)	31 (52.5%)		87 (40.1%)	25 (52.1%)		4 (44.4%)	1 (50.0%)		4 (36.4%)	1 (25.0%)		20 (55.6%)	4 (80.0%)	
Tumor size (mm)															
≤ 10.0	54 (19.8%)	4 (6.8%)	0.016	45 (20.7%)	4 (8.3%)	0.045	2 (22.2%)	0 (0.0%)	0.461	0 (0.0%)	0 (0.0%)		7 (19.4%)	0 (0.0%)	0.279
> 10.0	219 (80.2%)	55 (93.2%)		172 (79.8%)	44 (91.7%)		7 (77.8%)	2 (100.0%)		11 (100.0%)	4 (100.0%)	1.000	29 (80.6%)	5 (100.0%)	
Estrogen receptor															
Negative	50 (18.3%)	9 (15.3%)	0.577	3 (1.4%)	0 (0.0%)	0.413	0 (0.0%)	0 (0.0%)	1.000	–	–		–	–	–
Positive	223 (81.7%)	50 (84.7%)		214 (98.6%)	48 (100.0%)		9 (100.0%)	2 (100.0%)		–	–	–	–	–	
Progesterone receptor															
Negative	109 (39.9%)	21 (35.6%)	0.538	58 (26.7%)	11 (22.9%)	0.586	4 (44.4%)	1 (50.0%)	0.887	–	–	–	–	–	–
Positive	164 (60.1%)	38 (64.4%)		159 (73.3%)	37 (77.1%)		5 (55.6%)	1 (50.0%)		–	–		–	–	
Hormone receptor															
Negative	47 (17.2%)	9 (15.3%)	0.715	–	–	–	–	–	–	–	–	–	–	–	–
Positive	226 (82.8%)	50 (84.7%)		–	–		–	–		–	–		–	–	
HER2															
Negative	253 (92.7%)	53 (89.8%)	0.461	–	–	–	–	–	–	–	–	–	–	–	–
Positive	20 (7.3%)	6 (10.2%)		–	–		–	–		–	–		–	–	
Ki67															
≤ 14%	166 (60.8%)	40 (67.8%)	0.316	148 (68.2%)	37 (77.1%)	0.225	3 (33.3%)	0 (0.0%)	0.338	1 (9.1%)	0 (0.0%)	0.533	22 (61.1%)	3 (60.0%)	0.962
> 14%	107 (39.2%)	19 (32.2%)		69 (31.8%)	11 (22.9%)		6 (66.7%)	2 (100.0%)		10 (90.9%)	4 (100.0%)		14 (38.9%)	2 (40.0%)	
Lymphatic invasion															
ly0	201 (73.6%)	28 (47.5%)	< 0.001	158 (72.8%)	25 (52.1%)	0.005	7 (77.8%)	1 (50.0%)	0.425	8 (72.7%)	0 (0.0%)	0.013	28 (77.8%)	2 (40.0%)	0.074
ly1	72 (26.4%)	31 (52.5%)		59 (27.2%)	23 (47.9%)		2 (22.2%)	1 (50.0%)		3 (27.3%)	4 (100.0%)		8 (22.2%)	3 (60.0%)	
Venous invasion															
v0	264 (96.7%)	54 (91.5%)	0.073	209 (96.3%)	43 (89.6%)	0.051	9 (100.0%)	2 (100.0%)	1.000	10 (90.9%)	4 (100.0%)	0.533	36 (100.0%)	5 (100.0%)	1.000
v1	9 (3.3%)	5 (8.5%)		8 (3.7%)	5 (10.4%)		0 (0.0%)	0 (0.0%)		1 (9.1%)	0 (0.0%)		0 (0.0%)	0 (0.0%)	
Nuclear grade															
1, 2	243 (89.0%)	50 (84.7%)	0.356	203 (93.5%)	42 (87.5%)	0.151	9 (100.0%)	2 (100.0%)	1.000	7 (63.6%)	1 (25.0%)	0.185	24 (66.7%)	5 (100.0%)	0.125
3	30 (11.0%)	9 (15.3%)		14 (6.5%)	6 (12.5%)		0 (0.0%)	0 (0.0%)		4 (36.4%)	3 (75.0%)		12 (33.3%)	0 (0.0%)	
TILs (score)															
0, 1	219 (80.2%)	53 (89.8%)	0..082	190 (87.6%)	44 (91.7%)	0.423	7 (77.8%)	2 (100.0%)	0.461	5 (45.5%)	3 (75.0%)	0.310	17 (47.2%)	4 (80.0%)	0.169
2, 3	54 (19.8%)	6 (10.2%)		27 (12.4%)	4 (8.3%)		2 (22.2%)	0 (0.0%)		6 (54.5%)	1 (25.0%)		19 (52.8%)	1 (20.0%)	
TILs (score)															
0	12 (4.4%)	17 (28.8%)	< 0.001	11 (5.1%)	14 (29.2%)	< 0.001	0 (0.0%)	0 (0.0%)	1.000	0 (0.0%)	1 (25.0%)	0.086	1 (2.8%)	2 (40.0%)	0.003
1–3	261 (95.6%)	42 (71.2%)		206 (94.9%)	34 (70.8%)		9 (100.0%)	2 (100.0%)		11 (100.0%)	3 (75.0%)		35 (97.2%)	3 (60.0%)	

HER: human epidermal growth factor receptor. HR + HER2-BC: hormone receptor-positive and HER2 negative breast cancer (ER+ and/or PgR+, and HER2-). HR + HER2 + BC: hormone receptor-positive and HER2 positive breast cancer (ER+ and/or PgR+, and HER2+). HER2 enriched BC: human epidermal growth factor receptor 2-enriched breast cancer (ER-, PgR-, and HER2+). TNBC: triple negative breast cancer (ER-, PgR-, and HER2-). TILs: tumor- infiltrating lymphocytes

**Table 3:** Correlation between lymph node metastasis and clinicopathological features in cT1N0M0 breast cancer patients undergoing SLNB

Parameters	All intrinsic subtype (*n* = 319)			HR + HER2-BC (*n* = 255)			HR + HER2 + BC (*n* = 10)			HER2enriched BC (*n* = 14)			TNBC (*n* = 40)		
	pN0 or 1mic (*n* = 273)	pN1a or 2 (*n* = 46)	*p* value	pN0 or 1mic (*n* = 217)	pN1a or 2 (*n* = 38)	*p* value	pN0 (*n* = 9)	pN1a or 2 (*n* = 1)	*p* value	pN0 (*n* = 11)	pN1a or 2 (*n* = 3)	*p* value	pN0 (*n* = 36)	pN1a or 2 (*n* = 4)	*p* value
Age (years old)															
≤ 60	158 (57.9%)	21 (45.7%)	*0.124*	130 (59.9%)	17 (44.7%)	*0.081*	5 (55.6%)	1 (100.0%)	*0.389*	7 (63.6%)	2 (66.7%)	*0.923*	16 (44.4%)	1 (25.0%)	*0.455*
> 60	115 (42.1%)	25 (54.3%)		87 (40.1%)	21 (55.3%)		4 (44.4%)	0.(0.0%)		4 (36.4%)	1 (33.3%)		20 (55.6%)	3 (75.0%)	
Tumor size (mm)															
≤ 10.0	54 (19.8%)	3 (6.5%)	*0.017*	45 (20.7%)	3 (7.9%)	*0.062*	2 (22.2%)	0 (0.0%)	*0.598*	0 (0.0%)	0 (0.0%)	*1.000*	7 (19.4%)	0 (0.0%)	*0.332*
> 10.0	219 (80.2%)	43 (93.5%)		172 (79.3%)	35 (92.1%)		7 (77.8%)	1 (100.0%)		11 (100.0%)	3 (100.0%)		29 (80.6%)	4 (100.0%)	
Estrogen receptor															
Negative	50 (18.3%)	7 (15.2%)	*0.606*	3 (1.4%)	0 (0.0%)	*0.466*	0 (0.0%)	0 (0.0%)	*1.000*	–	–		–	–	
Positive	223 (81.7%)	39 (84.8%)		214 (98.6%)	38 (100.0%)		9 (100.0%)	1 (100.0%)		–	–		–	–	
Progesterone receptor															
Negative	109 (39.9%)	16 (34.8%)	*0.506*	58 (26.7%)	9 (23.7%)	*0.694*	4 (44.4%)	0 (0.0%)	*0.389*	–	–		–	–	
Positive	164 (60.1%)	30 (65.2%)		159 (73.3%)	29 (76.3%)		5 (55.6%)	1 (100.0%)		–	–		–	–	
Hormone receptor															
Negative	47 (17.2%)	7 (15.2%)	*0.735*	–	–		–	–		–	–		–	–	
Positive	226 (82.8%)	39 (84.8%)		–	–		–	–		–	–		–	–	
HER2															
Negative	253 (92.7%)	42 (91.3%)	*0.749*	–	–		–	–		–	–		–	–	
Positive	20 (7.3%)	4 (8.7%)		–	–		–	–		–	–		–	–	
Ki67															
≤ 14%	166 (60.8%)	30 (65.2%)	*0.567*	148 (68.2%)	28 (73.7%)	*0.500*	3 (33.3%)	0 (0.0%)	*0.490*	1 (9.1%)	0 (0.0%)	*0.588*	22 (61.1%)	2 (50.0%)	*0.667*
> 14%	107 (39.2%)	16 (34.8%)		69 (31.8%)	10 (26.3%)		6 (66.7%)	1 (100.0%)		10 (90.9%)	3 (100.0%)		14 (38.9%)	2 (50.0%)	
Lymphatic invasion															
ly0	201 (73.6%)	23 (50.0%)	*0.002*	158 (72.8%)	20 (52.6%)	*0.012*	7 (77.8%)	1 (100.0%)	*0.598*	8 (72.7%)	0 (0.0%)	*0.024*	28 (77.8%)	2 (50.0%)	*0.224*
ly1	72 (26.4%)	23 (50.0%)		59 (27.2%)	18 (47.4%)		2 (22.2%)	0 (0.0%)		3 (27.3%)	3 (100.0%)		8 (22.2%)	2 (50.0%)	
Venous invasion															
v0	264 (96.7%)	42 (91.3%)	*0.124*	209 (96.8%)	34 (89.5%)	*0.066*	9 (100.0%)	1 (100.0%)	*1.000*	10 (90.9%)	3 (100.0%)	*0.588*	36 (100.0%)	4 (100.0%)	*1.000*
v1	9 (3.3%)	4 (8.7%)		8 (3.7%)	4 (10.5%)		0 (0.0%)	0 (0.0%)		1 (9.1%)	0 (0.0%)		0 (0.0%)	0 (0.0%)	
Nuclear grade															
1, 2	243 (89.0%)	40 (87.0%)	*0.689*	203 (93.5%)	35 (92.1%)	*0.742*	9 (100.0%)	1 (100.0%)	*1.000*	7 (63.6%)	0 (0.0%)	*0.051*	24 (66.7%)	4 (100.0%)	*0.168*
3	30 (11.0%)	6 (13.0%)		14 (6.5%)	3 (7.9%)		0 (0.0%)	0 (0.0%)		4 (36.4%)	3 (100.0%)		12 (33.3%)	0 (0.0%)	
TILs (score)															
0, 1	219 (80.2%)	41 (89.1%)	*0.128*	190 (87.6%)	35 (92.1%)	*0.422*	7 (77.8%)	1 (100.0%)	*0.598*	5 (45.5%)	2 (66.7%)	*0.515*	17 (47.2%)	3 (75.0%)	*0.292*
2, 3	54 (19.8%)	5 (10.9%)		27 (12.4%)	3 (7.9%)		2 (22.2%)	0 (0.0%)		6 (54.5%)	1 (33.3%)		19 (52.8%)	1 (25.0%)	
TILs (score)															
0	12 (4.4%)	13 (28.3%)	*< 0.001*	11 (5.1%)	11 (28.9%)	*< 0.001*	0 (0.0%)	0 (0.0%)	*1.000*	0 (0.0%)	1 (33.3%)	*0.047*	1 (2.8%)	1 (25.0%)	*0.053*
1–3	261 (95.6%)	33 (71.7%)		206 (94.9%)	27 (71.1%)		9 (100.0)	1 (100.0%)		11 (100.0%)	2 (66.7%)		35 (97.2%)	3 (75.0)	

SLNB: sentinel lymph node biopsy. HER: human epidermal growth factor receptor. HR + HER2-BC: hormone receptor-positive and HER2 negative breast cancer (ER+ and/or PgR+, and HER2-). HR + HER2 + BC: hormone receptor-positive and HER2 positive breast cancer (ER+ and/or PgR+, and HER2+). HER2 enriched BC: human epidermal growth factor receptor 2-enriched breast cancer (ER-, PgR-, and HER2+). TNBC: triple negative breast cancer (ER-, PgR-, and HER2-). TILs: tumor- infiltrating lymphocytes

### Correlation between clinicopathological features and TILs

We examined the correlation between clinicopathological features and TILs in cN0 breast cancer cases (Table 4). When the patients were divided into TIL density score 0–1 and score 2–3, that is, a cut-off value of 10% was used for division into the higher group and lower group, the lower group correlated with the following clinicopathological factors; ER positive (p < 0.001), PgR positive (p < 0.001), HER2 negative (p = 0.013), Ki67 high (p = 0.002), nuclear grade high (p = 0.015). However, if the patients were divided into TIL density score 0 and score 1–3, that is, by the presence or absence of TIL density, correlation with these clinicopathological factors was not observed. When examined by intrinsic subtype, in HR + HER2-BC, patients with TILs density score 0 were significantly more aged (p = 0.035) and had a larger tumor size (p = 0.020) than in patients with TILs density score 1–3 (Supplementary Table 1). In HER2-enriched BC, the frequency of venous invasion was significantly higher in patients with TILs density score 0 than in patients with TILs density score 1–3 (p = 0.011). However, SLNM was significant in breast cancer with absent TIL density (p < 0.001). When examined by intrinsic subtypes, HR + HER-2 BC and HER2-enriched BC significantly correlated with SLNM, and TNBC also showed a similar tendency (HR + HER2-BC: p < 0.001, HER2-enriched BC: p = 0.047, TNBC: p = 0.053) (Table 3).

**Table 4:** Correlation between TILs and clinicopathological features in cT1N0M0 breast cancer patients undergoing SLNB

Parameters	tumor- infiltrating lymphocytes (*n* = 319)					
	Score 0 (*n* = 25)	Score 1–3 (*n* = 294)	*p* value	Score 0, 1 (*n* = 260)	Score 2, 3 (*n* = 59)	*p* value
Age (years old)						
≤ 60	10 (40.0%)	169 (57.5%)	*0.091*	144 (55.4%)	35 (59.3%)	*0.582*
> 60	15 (60.0%)	125 (42.5%)		116 (44.6%)	24 (40.7%)	
Tumor size (mm)						
≤ 10.0	1 (4.0%)	56 (19.0%)	*0.059*	49 (18.8%)	8 (13.6%)	*0.339*
> 10.0	24 (96.0%)	238 (81.0%)		211 (81.2%)	51 (86.4%)	
Estrogen receptor						
Negative	3 (12.0%)	54 (18.4%)	*0.425*	29 (11.2%)	28 (47.5%)	*< 0.001*
Positive	22 (88.0%)	240 (81.6%)		231 (88.8%)	31 (52.5%)	
Progesterone receptor						
Negative	9 (36.0%)	116 (39.5%)	*0.734*	88 (33.8%)	37 (62.7%)	*< 0.001*
Positive	16 (64.0%)	178 (60.5%)		172 (66.2%)	22 (37.3%)	
Hormone receptor						
Negative	3 (12.0%)	51 (17.3%)	*0.494*	27 (10.4%)	27 (45.8%)	*< 0.001*
Positive	22 (88.0%)	243 (82.7%)		233 (89.6%)	32 (54.2%)	
HER2						
Negative	24 (96.0%)	271 (92.2%)	*0.487*	245 (94.2%)	50 (84.7%)	*0.013*
Positive	1 (4.0%)	23 (7.8%)		15 (5.8%)	9 (15.35)	
Ki67						
≤ 14%	19 (76.0%)	177 (60.2%)		170 (65.4%)	26 (44.1%)	
> 14%	6 (24.0%)	177 (39.8%)	*0.119*	90.(34.6%)	33 (55.9%)	*0.002*
Lymphatic invasion						
ly0	19 (56.0%)	210 (71.4%)	*0.105*	182 (70.0%)	42 (71.2%)	*0.857*
ly1	11 (44.0%)	84 (28.6%)		78 (30.0%)	17 (28.8%)	
Venous invasion						
v0	25 (100.0%)	281 (95.6%)	*0.283*	252 (96.9%)	54 (91.5%)	*0.058*
v1	0 (0.0%)	13 (4.4%)		8 (3.1%)	5 (8.5%)	
Nuclear grade						
1, 2	24 (96.0%)	259 (88.1%)		236 (90.8%)	47 (79.7%)	
3	1 (4.0%)	35 (11.9%)	*0.230*	24 (9.2%)	12 (20.3%)	*0.015*
Pathological lymph node metastasis						
pN0 / pN1mic	12 (48.0%)	261 (88.8%)		219 (84.2%)	54 (91.5%)	
pN1a / pN2	13 (52.0%)	33 (11.2%)	*< 0.00121*	41 (15.8%)	5 (8.5%)	*0.150*

*TILs* tumor- infiltrating lymphocytes, *SLNB* sentinel lymph node biopsy, *HER* human epidermal growth factor receptor

**Fig. 2:**
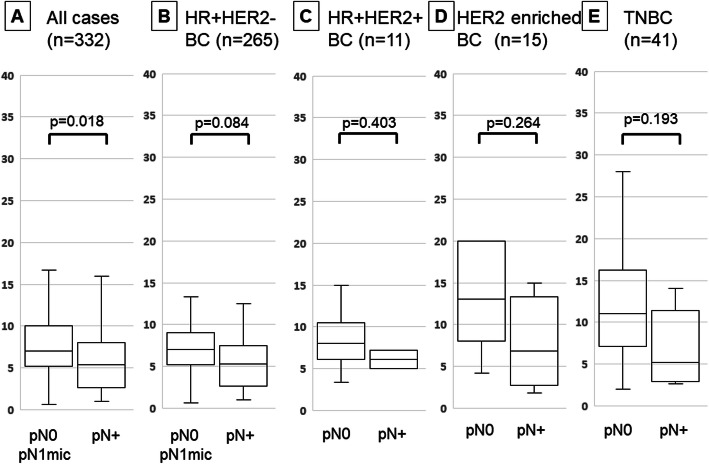
Comparison of tumor-infiltrating lymphocyte (TIL) density by differences in lymph node metastasis by box-plot diagrams in cT1 breast cancer: all (**a**), HR + HER2-BC (**b**), HR + HER2 + BC (**c**), HER2-enriched BC (**d**), triple-negative breast cancer (**e**). Correlation was performed by Student’s t test

**Fig. 3:**
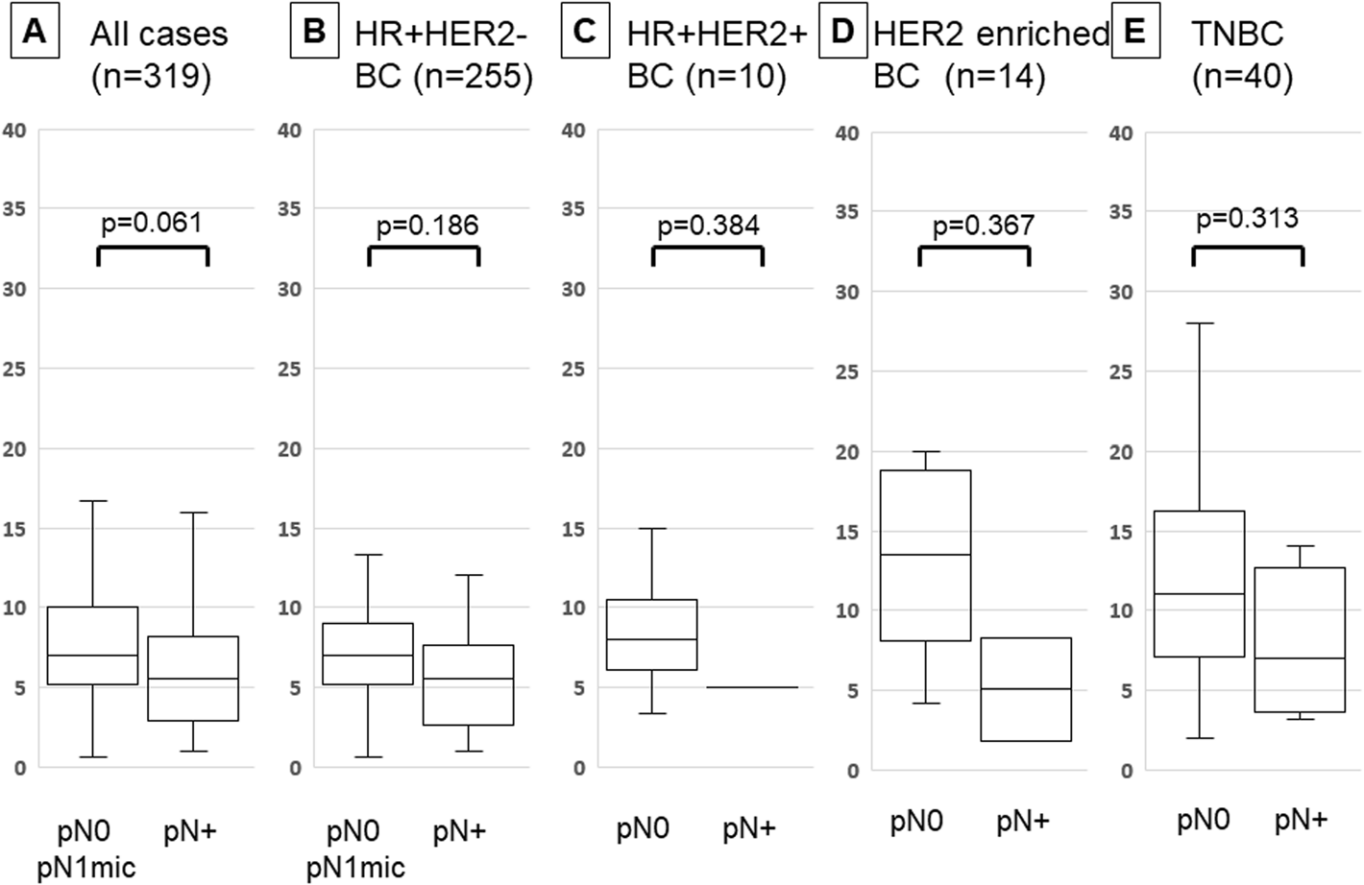
Comparison of tumor-infiltrating lymphocyte (TIL) density by differences in lymph node metastasis by box-plot diagrams in cT1N0M0 breast cancer patients undergoing SLNB: all (**a**), HR + HER2-BC (**b**), HR + HER2 + BC (**c**), HER2-enriched BC (**d**), triple-negative breast cancer (**e**). Correlation was performed by Student’s t test

TIL density was significantly lower in patients with lymph node metastasis than in those without it in all cT1 patients (p = 0.018) (Fig. 2). When examined by intrinsic subtype, there was no significant difference between the subtypes. Moreover, no significant difference was observed in all cases when focusing on cN0 cases (p = 0.061) (Fig. 3).

Based on these results, multivariate analysis for SLNM predictors revealed that lymphatic invasion (p = 0.008, OR = 2.522) and TILs (p < 0.001, OR = 0.137) were independent factors for prediction of SLNM (Table 5).

**Table 5:** Univariate and multivariate analysis with sentinel lymph node metastasis for cT1N0M0 breast cancer

Parameters	Univarite analysis			Multivarite analysis		
	Odd ratio	95% CI	*p* value	Odd ratio	95% CI	*p* value
Age at operation (years old) ≤ 60 vs > 60	1.636	0.873–3.065	0.124			
Tumor size (mm) ≤ 10.0 vs > 10.0	3.534	1.056–11.825	0.017	2.639	0.888–11.346	0.085
Estrogen receptor Negative vs Positive	1.249	0.528–2.955	0.606			
Progesterone receptor Negative vs Positive	1.246	0.648–2.395	0.506			
Hormone receptor Negative vs Positive	1.159	0.488–2.748	0.735			
HER2 Negative vs Positive	1.205	0.392–3.700	0.749			
Ki67 ≤ 14% vs > 14%	0.827	0.430–1.590	0.567			
Lymphatic invasion ly0 vs ly1	2.792	1.476–5.282	0.002	2.522	1.280–4.973	0.008
Venous invasion v0 vs v1	2.794	0.823–9.481	0.124			
Nuclear grade 1, 2 vs 3	1.215	0.475–3.105	0.689			
TILs 0, 1 vs 2, 3	0.495	0.187–1.311	0.128			
TILs 0 vs 1–3	0.117	0.049–0.277	< 0.001	0.137	0.055–0.335	< 0.001

*CI* confidence intervals, *HER2* human epidermal growth factor receptor 2, *TILs* tumor- infiltrating lymphocytes

## Discussion

Numerous studies have reported predictors of SLNM. Although some studies have reported age [6–9], site [6, 10, 24], ER positivity [7, 24], PgR positivity [8, 24], HER2 positivity [25] as predictors of SLNM, the most commonly reported predictors are tumor size [6–10, 24, 25], lymphatic invasion [6–8, 24, 25], and pathological nuclear grade [6–10, 24, 25]. In our study, the SLNM rate was similar to previous reports, and tumor size and lymphatic invasion were found to be predictive factors. However, intrinsic subtype and nuclear grade were not found to be predictors in our study. In recent years, it has been known that the pathological response to preoperative chemotherapy is a predictor of prognosis [26–29]. Based on these reports, preoperative chemotherapy is actively administered in HER2-positive breast cancer and TNBC because the treatment response is greater than that in hormone receptor-positive breast cancer. As a result, the number of patients who underwent surgery primarily for HER2-positive breast cancer or TNBC was considered to be the reason for conducting this study.

After defining the cut-off value for TIL density as 10%, as previously reported, hormone-positive breast cancer was observed to have lower TIL density while hormone-negative breast cancer or HER2-positive breast cancer were observed to have higher TIL density in this study [13, 14]. When the correlation between TILs and clinicopathological factors was examined, in HR + HER2-BC, the correlations between TILs and tumor size or age were shown. Regarding the tumor size, it has recently been reported that the microenvironment around the cancer changes depending on the local progression [30]. According to the report, not only CD8 + lymphocytes that suppress cancer progression but also FOXP3-positive lymphocytes that promote cancer progression are reduced. In other words, as cancer progresses, immune escape may begin to occur, and metastases are likely to occur accordingly. Regarding age, we have previously reported that young breast cancer patients tend to have higher TILs density (date not shown). That may have influenced the results in this time. This study suggests that the tumor immune-microenvironment is involved in lymph node metastasis. Our hypothesis was that the TIL density may be a predictor of SLNM. The correlation between TILs and lymph node metastasis has been reported in gastric cancer, melanoma, and breast cancer [31–33]. A study on breast cancer examined 76 patients who underwent surgery first and 96 patients who underwent preoperative chemotherapy, and it reported that there was a correlation between TILs and lymph node metastasis in both groups. Interestingly, Caziuc evaluated not only SLNs but also axillary lymph nodes in cases of additional axillary lymph node dissection due to SLNM. However, detailed analysis of the subtypes that could affect TIL density was not conducted, and no detailed data were provided on the relationship between TILs and clinicopathological factors. Furthermore, no relationship was found between any clinicopathological features other than TILs and lymph node metastasis. Accordingly, this report did not examine clinicopathological factors other than TILs, which are predictors of lymph node metastasis. However, our research is significant because we examined the correlation between TILs and clinicopathological factors such as all the subtypes and performed multivariate analysis to determine the predictors of SLNM, including TILs.

We are aware that our study has some limitations. Firstly, there were few HER2-positive breast cancer and TNBC patients, as we have stated earlier. Furthermore, there were a few cases with distant metastases along with a primary lesion of less than 20 mm that were excluded from our study. However, some studies have reported that TIL density is predictive of chemotherapy response [34, 35]. Therefore, if SLNB was omitted even if the SLN had metastasized in cN0 breast cancer with high TIL density, postoperative chemotherapy would be expected to have a high therapeutic effect and not affect the prognosis.

## Conclusions

Our study suggests a correlation between lymph node metastasis and the tumor immune-microenvironment in cT1 breast cancer cases. Moreover, TIL density may be a predictor of SLNM in breast cancer patients without lymph node metastasis on preoperative imaging.

## Supplementary information

**Additional file 1: Supplementary Table 1.** Correlation between TILs and clinicopathological features in cT1N0M0 breast cancer patients undergoing SLNB by intrinsic subtype.

## Data Availability

The datasets used and/or analyzed during the current study are available from the corresponding author on reasonable request.

## Abbreviations

BC: Breast cancer
CI: Confidence intervals
CT: Computed tomography
ER: Estrogen receptor
HER2: Human epidermal growth factor receptor 2
HR: Hormone receptor
OR: Odds ratio
PgR: Progesterone receptor
SLN: Sentinel lymph node
SLNB: Sentinel lymph node biopsy
SLNM: Sentinel lymph node metastasis
TILs: Tumor-infiltrating lymphocytes
TNBC: Triple-negative breast cancer
US: Ultrasonography
VAB: Vacuum-assisted biopsy
