# Supplementary material for: Prediction of lymph node metastasis by tumor-infiltrating lymphocytes in T1 breast cancer
Source: BMC Cancer. 2020 Jun 26;20:598. doi: 10.1186/s12885-020-07101-y (PMC7318528; doi:10.1186/s12885-020-07101-y)
Supplement: Supplementary file 1 — Additional file 1: Supplementary Table 1. Correlation between TILs and clinicopathological features in cT1N0M0 breast cancer patients undergoing SLNB by intrinsic subtype. [file 12885_2020_7101_MOESM1_ESM.docx]

**Supplementary Table 1. Correlation between TILs and clinicopathological features in cT1N0M0 breast cancer patients undergoing SLNB by intrinsic subtype**

| Parameters | All intrinsic subtype (*n* = 319) | | | HR+HER2-BC (*n* =255) | | | HR+HER2+BC (*n* =10) | | | HER2enriched BC (*n* =14) | | | TNBC (*n* =40) | | |
| --- | --- | --- | --- | --- | --- | --- | --- | --- | --- | --- | --- | --- | --- | --- | --- |
|  | Score 0  (n =25) | Score 1-3  (n = 294) | *p* value | Score 0  (n =22) | Score 1-3  (n = 233) | *p* value | Score 0  (n =0) | Score 1-3  (n = 10) | *p* value | Score 0  (n =1) | Score 1-3  (n = 13) | *p* value | Score 0  (n =2) | Score 1-3  (n = 38) | *p* value |
| Age (years old)  ≤ 60  > 60 | 10 (40.0 %)  15 (60.0 %) | 169 (57.5 %)  125 (42.5 %) | 0.091 | 8 (36.4 %)  14 (63.6 %) | 139 (59.7 %)  94 (40.3 %) | 0.035 | 0 (0.0 %)  0 (0.0 %) | 6 (60.0 %)  4 (40.0 %) | - | 0 (0.0 %)  1 (100.0 %) | 8 (61.5 %)  5 (38.5 %) | 0.231 | 1 (50.0 %)  1 (50.0 %) | 16 (42.1 %)  22 (57.9 %) | 0.826 |
| Tumor size (mm)  ≤ 10.0  > 10.0 | 1 (4.0 %)  24 (96.0 %) | 56 (19.0 %)  238 (81.0 %) | 0.059 | 1 (4.5 %)  22 (95.5 %) | 47 (20.2 %)  186 (79.8 %) | 0.020 | 0 (0.0 %)  0 (0.0 %) | 2 (20.0 %)  8 (80.0 %) | - | 0 (0.0 %)  1 (100.0 %) | 0 (0.0 %)  13 (100.0 %) | 1.000 | 0 (0.0 %)  2 (100.0 %) | 7 (18.4 %)  31 (81.6 %) | 0.504 |
| Estrogen receptor  Negative  Positive | 3 (12.0 %)  22 (88.0 %) | 54 (18.4 %)  240 (81.6 %) | 0.425 | 0 (0.0 %)  22 (100.0 %) | 3 (1.3 %)  230 (98.7 %) | 0.592 | 0 (0.0 %)  0 (0.0 %) | 0 (0.0 %)  10 (100.0 %) | - | -  - | -  - |  | -  - | -  - |  |
| Progesterone receptor  Negative  Positive | 9 (36.0 %)  16 (64.0 %) | 116 (39.5 %)  178 (60.5 %) | 0.734 | 6 (27.3 %)  16 (72.7 %) | 61 (26.7 %)  172 (73.8 %) | 0.911 | 0 (0.0 %)  0 (0.0 %) | 4 (40.0 %)  6 (60.0 %) | - | -  - | -  - |  | -  - | -  - |  |
| Hormone receptor  Negative  Positive | 3 (12.0 %)  22 (88.0 %) | 51 (17.3 %)  243 (82.7 %) | 0.494 | -  - | -  - |  | -  - | -  - |  | -  - | -  - |  | -  - | -  - |  |
| HER2  Negative  Positive | 24 (96.0 %)  1 (4.0 %) | 271 (92.2 %)  23 (7.8 %) | 0.487 | -  - | -  - |  | -  - | -  - |  | -  - | -  - |  | -  - | -  - |  |
| Ki67  ≤14 %  >14 % | 19 (76.0 %)  6 (24.0 %) | 177 (60.2 %)  117 (39.8 %) | 0.119 | 18 (81.8 %)  4 (18.2 %) | 158 (67.8 %)  75 (32.2 %) | 0.174 | 0 (0.0 %)  0 (0.0 %) | 3 (30.0 %)  7 (70.0 %) | - | 0 (0.0 %)  1 (100.0 %) | 1 (0.0 %)  12 (92.3 %) | 0.773 | 1 (50.0 %)  1 (50.0 %) | 15 (39.5 %)  23 (60.5 %) | 0.767 |
| Lymphatic invasion  ly0  ly1 | 19 (56.0 %)  11 (44.0 %) | 210 (71.4 %)  84 (28.6 %) | 0.105 | 13 (59.1 %)  9 (40.9 %) | 165 (70.8 %)  68 (29.2 %) | 0.252 | 0 (0.0 %)  0 (0.0 %) | 8 (80.0 %)  2 (20.0 %) | - | 0 (0.0 %)  1 (100.0 %) | 8 (61.5 %)  5 (38.5 %) | 0.231 | 1 (50.0 %)  1 (50.0 %) | 29 (76.3 %)  9 (23.7 %) | 0.402 |
| Venous invasion  v0  v1 | 25 (100.0 %)  0 (0.0 %) | 281 (95.6 %)  13 (4.4 %) | 0.283 | 22 (100.0 %)  0 (0.0 %) | 221 (94.8 %)  12 (5.2 %) | 0.276 | 0 (0.0 %)  0 (0.0 %) | 10 (100.0 %)  0 (0.0 %) | - | 0 (0.0 %)  1 (100.0 %) | 12 (92.3 %)  1 (7.7 %) | 0.011 | 2 (100.0 %)  0 (0.0 %) | 38 (100.0 %)  0 (0.0 %) | 1.000 |
| Nuclear grade  1, 2  3 | 24 (96.0 %)  1 (4.0 %) | 259 (88.1 %)  35 (11.9 %) | 0.230 | 22 (100.0 %)  0 (0.0 %) | 216 (92.7 %)  17 (7.3 %) | 0.190 | 0 (0.0 %)  0 (0.0 %) | 10 (100.0 %)  0 (0.0 %) | - | 0 (0.0 %)  1 (100.0 %) | 7 (53.8 %)  6 (46.2 %) | 0.299 | 2 (100.0 %)  0 (0.0 %) | 26 (68.4 %)  12 (31.6 %) | 0.342 |
| Pathological lymph node metastasis  pN0 / pN1mic  pN1a / pN2 | 12 (48.0 %)  13 (52.0 %) | 261 (88.8 %)  33 (11.2 %) | <0.001 | 11 (50.0 %)  11 (50.0 %) | 206 (88.4 %)  27 (11.6 %) | <0.001 | 0 (0.0 %)  0 (0.0 %) | 9 (90.0 %)  1 (10.0 %) | - | 0 (0.0 %)  1 (100.0 %) | 11 (84.6 %)  2 (15.4 %) | 0.047 | 1 (50.0 %)  1 (50.0 %) | 35 (92.1 %)  3 (7.9 %) | 0.053 |

SLNB: sentinel lymph node biopsy. HER: human epidermal growth factor receptor. HR+HER2-BC: hormone receptor-positive and HER2 negative breast cancer (ER+ and/or PgR+, and HER2-). HR+HER2+BC: hormone receptor-positive and HER2 positive breast cancer (ER+ and/or PgR+, and HER2+). HER2 enriched BC: human epidermal growth factor receptor 2-enriched breast cancer (ER-, PgR-, and HER2+). TNBC: triple negative breast cancer (ER-, PgR-, and HER2-). TILs: tumor- infiltrating lymphocytes.
